# Social stress increases the susceptibility to infection in the ant *Harpegnathos saltator*

**DOI:** 10.1038/srep25800

**Published:** 2016-05-10

**Authors:** Sebastian A. Schneider, Charlotte Scharffetter, Anika E. Wagner, Christine Boesch, Iris Bruchhaus, Gerald Rimbach, Thomas Roeder

**Affiliations:** 1 Kiel University, Zoology, Dept. Molecular Physiology, Kiel, Germany; 2 Kiel University, Food Sci Res Group, Kiel, Germany; 3 Bernhard-Nocht-Institute for Tropical Medicine, Hamburg, Germany; 4 Airway Research Center North (ARCN), Members of the German Center for Lung Research (DZL), Germany

## Abstract

Aggressive interactions between members of a social group represent an important source of social stress with all its negative follow-ups. We used the ponerine ant *Harpegnathos saltator* to study the effects of frequent aggressive interactions on the resistance to different stressors. In these ants, removal or death of reproducing animals results in a period of social instability within the colony that is characterized by frequent ritualized aggressive interactions leading to the establishment of a new dominance structure. Animals are more susceptible to infections during this period, whereas their resistance against other stressors remained unchanged. This is associated with a shift from glutathione-S-transferase activities towards glutathione peroxidase activities, which increases the antioxidative capacity at the expense of their immune competence.

Stress is usually defined as an alteration that perturbs organismic homeostasis. In order to re-establish a homeostatic equilibrium, organisms are able to launch stress responses^1^,^2^. Strong and prolonged confrontation with stressors is believed to be negatively correlated with the immune system’s performance and therewith with the resistance towards infections^3^,^4^. Although the existence of this relationship between stress and immunity is commonly accepted, the underlying molecular mechanisms are not fully understood^5^,^6^.

Stress can be physical or behavioural in nature. Behaviourally induced stress results from interactions with conspecifics and individuals from other species, primarily with potential predators and with prey. In animals living in social groups, stress induced by interactions with conspecifics is more important than in animals with a different life style. Social stress comes in different flavours, ranging from very subtle forms of interactions up to highly aggressive encounters. To modulate the immune response, the information content of behavioural stress has to be transmitted from the nervous to the immune system. In mammals, this translation of behavioural stress into a modified immune response is mediated via the major hormone axes involving glucocorticoids and the sympathetic nervous system^7^. This type of interaction between stress and the immune system is not restricted to higher vertebrates, as even insect immunity critically reacts to a number of potential stressors indicating that this relationship is phylogenetically very ancient^8^.

So far, mostly rodents were used as model organisms to study the outcome of behavioural stress on health or the immune status. Other models have almost completely been neglected. However, social stress occurs in other systems as well, notably in eusocial insects that live in large colonies where reproduction is restricted to few individuals in the colony while the rest, usually termed workers, helps to raise the offspring^9^. Although most of the colony life in the majority of insect societies is seemingly harmonic, some species are characterized by open reproductive conflicts among colony members, which is most likely associated with social stress and can have an impact on immune function^10^.

Eusocial insect species with a high reproductive potential of workers are prone to develop open reproductive conflicts. This is characteristic for many ponerine ant species, where workers can actually replace queens as reproductive individuals^11^. When the queen dies, many workers start fighting over queen succession. In the ponerine ant, *Harpegnathos saltator*, the period of queen replacement is characterized by three types of aggressive interaction: i) duelling is mutual antennation between potential successors, ii) domination of subordinates through biting, and iii) policing of potential successors by subordinate nestmates through grabbing and dragging. After weeks of fighting, some individuals establish themselves as reproductive workers, so-called gamergates^11^.

The transition from worker to gamergate is accompanied by profound behavioural and physiological changes. Gamergates are aggressive towards own nestmates but are timid towards intruders while non-reproductive workers are highly aggressive towards foreign individuals, while being submissive towards gamergates. Activation of their ovaries and regular egg-laying is associated with changes in the cuticular pheromone profile that signals their reproductive status. In addition, the transition towards a reproductive is associated with an at least duplication of their lifespan. However, the only way to reach the status of the gamergate is to go through the period of intense and ritualized aggression^12^,^13^.

We used this unique system to study the effects of social instability and increased aggression between nestmates on stress resistance and susceptibility towards infections and correlated this with the expression and presence of enzymatic and non-enzymatic antioxidants. For this, we employed two representative major stressors, namely infection (with the insect pathogen *Erwinia carotovora*)^14^ and oxidative stress (injection of the superoxide anions producing compound paraquat). Those ants actively engaged in aggressive interactions showed an increased susceptibility to infections, whereas their resistance to oxidative stress remained almost unchanged. In parallel, these ants shifted an important aspect of their glutathione metabolism from glutathione-S-transferase towards glutathione peroxidase activities, reflecting a shift from the detoxification of electrophilic endogenous and xenobiotic compounds towards a higher peroxidase activity to detoxify lipid hydroperoxides and free hydrogen peroxides.

## Results

**Figure 1:**
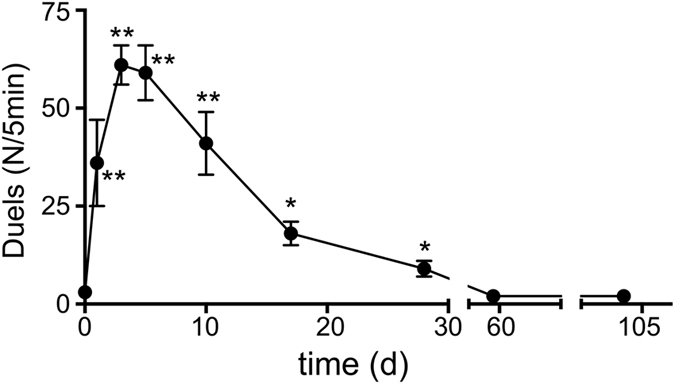
Time course of aggressive interactions following removal of reproductive animals. All reproductive animals were taken from an experimental colony and the aggressive interactions between nestmates were counted at different time points following removal of the gamergates (n = 20, statistically significant differences are marked by asterics; *p <  0.05, **p < 0.01, ***p < 0.005).

Colonies of the ponerine ant *Harpegnathos saltator* can undergo a secondary polygyny. After death of the queen or the sexual reproductive gamergates, the colonies undergo a period of social instability, which is characterized by aggressive interactions between nestmates leading to the establishment of a new social hierarchy, meaning that dominant workers develop into reproductive animals, called gamergates^11^. This period of social instability is characterized by a significant increase of aggressive interactions between nestmates. Usually, this aggressive behaviour peaks after 6–8 days, but the increase in aggressive behaviour prolongs up to a period of 3 to 4 weeks (Fig. 1).

**Figure 2:**
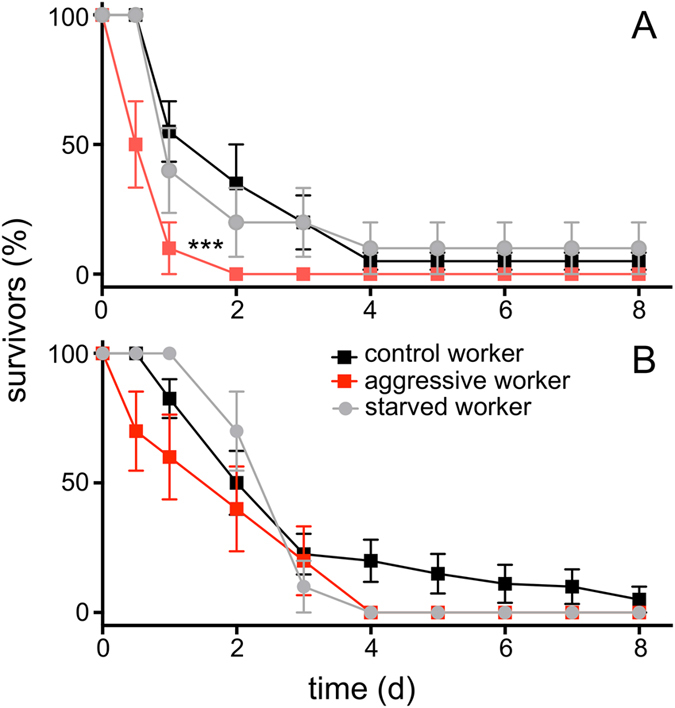
Survival of ants following injection of stressors. Fixed amounts of *Erwinia carotovora* (**A**) or paraquat (**B**) were injected in different types of ants. Control workers (black) and workers engaged in aggressive interactions during the period of social instability were used (red) or control workers starved for 24 h (grey; N = 20 for all experiments. Data points ± standard deviations are given; ***indicates statistically different curves p < 0.005).

Workers engaged in aggressive interactions were selected for our induced stress experiment during these periods of social instability. These workers showed a significantly increased susceptibility towards an experimental infection with the insect pathogen *Erwinia carotovora* (Fig. 2A). We used injection of bacteria instead of natural ways of infection, because this protocol induced death with a much greater reproducibility. Control animals (taken from a colony with a stable, unmanipulated dominance structure) injected with *E. carotovora* survived longer (median survival time 2.5 days) than those taken from colonies within a period of social instability (median survival time 1 day). An almost linear decline was observed up to the 4^th^ day following injection and some animals were still alive at the end of the experiment. In contrast, most animals taken from the colonies experiencing social instability died during the first day and none of them survived longer than two days (Fig. 2A). This difference was statistically highly significant (df = 1, n = 20 animals per group, log rank analysis, chi^2^ = 22.73, Hazard ratio = 0.36, p < 0.0001). We never observed aggressive behaviours of nestmates against treated animals that might have increased their chance of dying. As an additional control, we starved animals for the same time period to exclude confounding effects associated with limited energy uptake that may occur during this period. These individuals showed survival rates indistinguishable from those of the normal workers (Fig. 2A).

Injection of paraquat instead of bacteria mimics strong oxidative stress (production of superoxide anions). We compared the resistance of control ants and those from socially unstable colonies with aggressive workers towards paraquat injection. Here, the aggressive animals show a slightly higher susceptibility (median survival time of control workers treated with paraquat 2.5 days, *vs* 2 days for aggressive workers), which is nevertheless statistically not significant (df = 1, n = 20 animals per group, log rank analysis, chi^2^ = 2.34, Hazard ratio = 0.63, p = 0.128; Fig. 2B). As observed in the infection experiments, injection of paraquat into starved workers gave curves that are statistically undistinguishable from the control ones (Fig. 2B). A power analysis revealed that at least 80–100 animals per group would have been necessary to show differences between both groups.

### Enzymatic and nonenzymatic antioxidants

**Figure 3:**
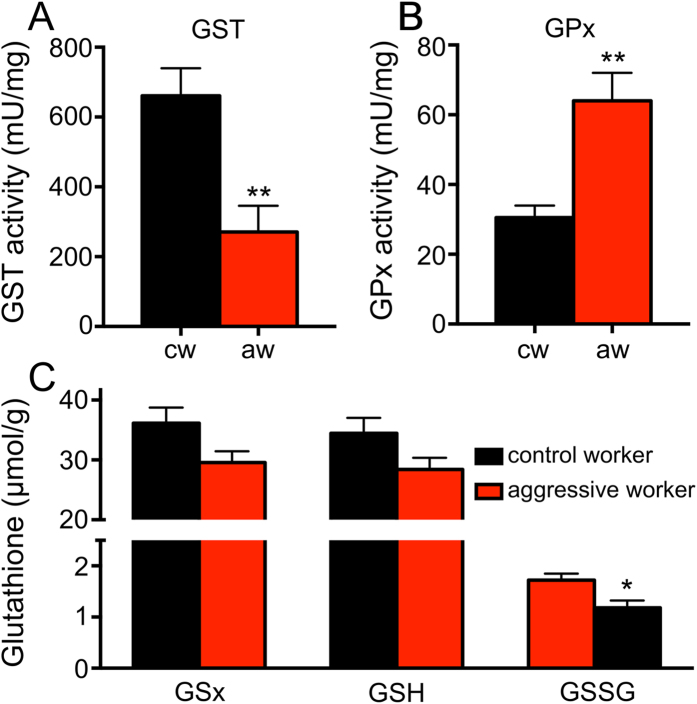
Enzyme activities and glutathione levels in control workers (black) and those engaged in aggressive interactions (red). In (**A**), the enzyme activities of glutathione-S-transferases and in (**B**) those of glutathione peroxidases are displayed. In (**C**), concentrations of glutathione derivatives are shown. Total glutathione concentrations (GSx; left), reduced glutathione concentrations (GSH; middle) and oxidized glutathione concentrations (GSSG, glutathione disulfide; right) are listed. cw means control worker, aw means aggressive workers. All experiments were performed with 10 animals each, mean values ± standard deviations are displayed, indication of statistical significance see Fig. 1.

Comparing the levels of enzymatic antioxidants revealed a pronounced difference between control workers and those taken from colonies characterized by social instability and enhanced aggression. Two enzyme systems that are important for the glutathione metabolism show changes in their activities that point into opposite directions. These are glutathione-S-transferase (GST) and glutathione peroxidase activities (GPx). As the total GST activity of workers engaged in aggressive interactions is almost 60% lower than that of control workers, the corresponding GPx activities are more than twofold higher in these workers compared with matching controls (Fig. 3A,B). These differences are highly significant (3A; df = 20, t = 3.51, p = 0.003 for GST), (3B; df = 20, t =  3.84, p = 0.0012 for GPx). Moreover, we tested if the animals taken from the two different categories (control and workers engaged in aggressive interactions) differed in their total glutathione levels, their levels of reduced and oxidized glutathione and their ratio between oxidized and reduced glutathione (Fig. 3C). All three groups of glutathione are present in lower amounts in workers engaged in aggressive interactions and this difference is statistically significant for the oxidized form of glutathione (glutathione disulphide, GSSG, df = 20, unpaired t-test, t = 2.81, p = 0.012). For total glutathione levels and those of reduced glutathione these differences are not statistically significant (GSx, df = 20, unpaired t-test, t = 2.05, p = 0.055; GSH, df = 20, unpaired t-test, t = 1.86, p = 0.079). The differences in the ratios between oxidized and reduced glutathione (GSH/GSSG) is also not altered with 95% GSH and 5% GSSG for normal workers and 95,85% GSH and 4.15% GSSG for aggressive workers.

**Figure 4:**
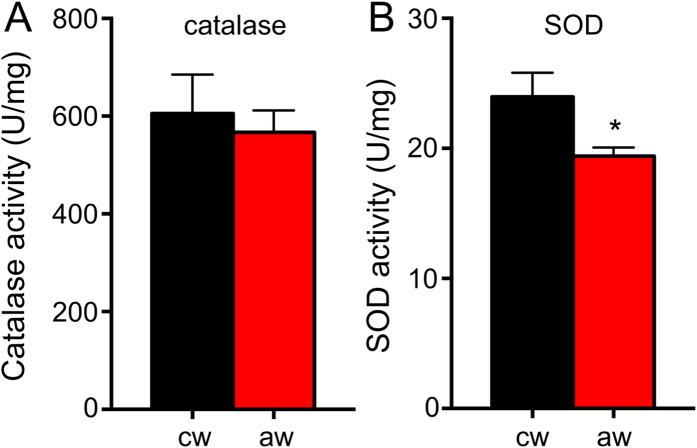
Catalase and SOD activities. In (**A**) catalase and in (**B**) SOD activities are given. cw means control worker, aw means aggressive workers. All experiments were performed with 10 animals each, mean values ± standard deviations are displayed, statistics see Fig. 1.

In addition, we tested the activities of two major antioxidant enzyme activities, namely those of catalase and SOD (Fig. 4). While catalase activities are unaltered (Fig. 4A; df = 20, unpaired t-test, t = 0.42, p = 0.68). SOD activities are slightly lower in workers engaged in aggressive encounters if compared with control workers (Fig. 4B; df = 20, unpaired t-test, t = 2.32, p = 0.032).

## Discussion

Workers of the ponerine ant *Harpegnathos saltator* that were actively engaged in aggressive interactions showed reduced survival rates after infection with the pathogenic bacterium *Erwinia carotovora*. In contrast, injection of the superoxide anions producing compound paraquat, induced mortality was not increased in ants engaged in aggressive encounters, implying that aggression and social stress specifically reduces resistance to infection, while resistance to other stressors is not changed. Moreover, glutathione-S-transferase (GST) activities were substantially decreased in aggressive workers, while glutathione peroxidase (GPx) activities were significantly enhanced in these animals suggesting that an enzymatic shift occurs presumably to cope with higher oxidative stress.

The most important finding of this study that social stress reduces the resistance against an infection, is not restricted to this ant species, but may be common to all animals living in a social context. The first report about a correlation between social stress and disease resistance was published almost a century ago, where the tuberculosis incidence of Japanese school children was correlated with stress^15^. This correlation between stress and susceptibility to infection was shown several time e.g. in human subjects that were experimentally exposed to respiratory viruses, where the correlation between social stress and the probability for disease develop was shown^16^. In HIV-patients, severity of disease development was correlated with cumulative social stress indicators^17^. Moreover, similar correlations between stress, the hormonal system and the immune response have been reported in several insect models^18^,^19^.

The mechanistic link between stress and the immune system is not fully understood, but stress induced hormones appear to take this^20^. In vertebrates, this is primarily mediated by the HPA-axis, comprising the adrenergic system as terminally active effector hormones. Insects on the other hand appear to have a less complex system with octopamine and tyramine, the invertebrate counterparts of epinephrine and norepinephrine acting as the major hormones^21^,^22^,^23^. This insect adrenergic system appears to link the inhibitory effects of high stress levels with immunity^4^. Another possible explanation for the enhanced susceptibility towards infection is the shift in the hormonal profile of the ants that is associated with developing into reproductive animals^24^. Previously, we could show that corresponding animals, either those that are already reproductive (gamergates) or those that are on the track to develop into reproductive (those that have been isolated for a short period of time), show an enhanced resistance instead of an enhanced susceptibility towards infection^12^, which would be contradictory to this assumption.

Surprisingly, only the increased susceptibility towards an infection under this type of social stress was statistically significant. The effects of paraquat (oxidative stress or more precisely the production of superoxide anions) on the other hand showed the same trend, but without being statistically significant. We didn’t observe this differential susceptibility in response to these two stressors in a previous experimental paradigm were *Harpegnathos* workers and gamergates were compared^12^. If we compare workers with reproductive gamergates or with workers that have been isolated for a short period, the observed differences in stress resistance applied for both types of stressors, oxidative stress and infection. Comparing the levels of antioxidants and the levels of enzymatic antioxidant activities between these two types of experiments (worker *vs* gamergates and workers *vs* isolated workers) revealed strongly decreased catalase activities and decreased glutathione levels in the more resistant ants^12^. Workers engaged in aggressive interactions on the other side didn’t show these dramatic changes, although a slight decrease in catalase activities could be observed. Instead, they show a very intriguing and reciprocal shift in two enzymatic activities of the glutathione metabolism. Glutathione-*S*-transferase activities are significantly lower while glutathione peroxidase activities are significantly higher in these aggressive ants. As GSTs and GPx have partially overlapping functions, these results are not easy to explain^25^. GPx activity detoxifies superoxide moieties using glutathione as a substrate, thus functionally replacing catalases. In these animals, an increased ability to detoxify superoxide radicals may be paid for by a dramatically decreased GST activity. This reallocation of enzymatic activities may be part of a stress-induced larger reconfiguration of the animal’s physiology^26^. Nevertheless, the reduced GST-activity is one possible reason for the strongly reduced ability to fight infections. Usually, upregulation of GST-expression is a major response towards infection in insects^27^. Nevertheless, it has to be mentioned that the normal role of GSTs is to detoxify e.g. peroxidised lipids or xenobiotics. Thus, it appears that social instability and aggression generally decreases stress resistance in these animals. At the same time, a presumably hormonally driven reallocation of enzymatic activities leads to the situation that resistance towards infection is reduced, while this is not the case for resistance towards other stressors, such as oxidative stress. The shift in the glutathione metabolism is one potential mechansim responsible for this. This reallocation appears to represent a way to cope with these aggressive interactions, as they occur primarily within the hive and during this period of social instability, ants tend not to leave the hive, which would probably decrease the risk of exposure to new pathogens.

## Methods

### Colony maintenance and experimental groups

Individual ants for all experimental procedures were taken from gamergate-right colonies. These colonies were collected as gamergate-right colonies in the years 1994, 1995 and 1999 in the area of Jog Falls in Karnataka State, South India and kept under laboratory conditions as inbred colonies since then. The colonies were housed in plastic boxes (19 cm × 19 cm × 10 cm) containing a floor of plaster with a carved out nest chamber covered with a glass plate. The colonies were provided with live crickets *ad libitum* and housed at 25 °C under a 12h/12h light/dark regime^12^. Control ants were taken from fully reproductive colonies that fulfilled the following criteria: i) the minimum size of the colony was 80 ants, ii) the colonies contained eggs, larvae and pupae, iii) the colonies showed an established and stable dominance hierarchy verified by inconspicuous levels of aggression during a fixed regime of observations (12 observation periods lasting 5 min each within 24 h performed several times with less than 5 duels per observation period). The experimental colonies (starting from those characterized above) were generated by removal of all reproductive animals from the colony.

### Sampling strategy

To exclude colony dependent biases, we used a matching sample strategy. At the same time, this strategy ensures that a population covering genetic diversity is reflected in the experiments. For survival experiments, we took a total of 20 animals for each experimental group (control and treated) from 10 different colonies (2 animals from each colony). Before induction of aggressive behaviors, all animals of an experimental colony were labeled with an individual color code using enamel colors. This coloring had been tested before the onset of the experiments and it induced no changes in the animals’ behavior even over longer observation periods. Control animals were subjected to the same handling procedures. Aggressive workers were isolated following removal of gamergates from a colony. Within 24 hours after removal of the gamergates ritualized, aggressive interactions between workers start. Following induction of dominance fights, the colony was monitored 9 times for 5 minutes each in a period of 4h. Only those workers that were engaged at least in 5 out of the 9 observation periods in aggressive interactions (dueling) were scored as aggressive workers and taken for our experiments (the time point of removal from the colonies was thus 28 h–48 h after removal of gamergates).

### Survival assays - Injection procedure

The animals were immobilized by keeping them on ice for a few minutes. The ants were then injected with 40 nl of medium using a sharp glass capillary (either containing the stressor or pure medium as a control) into the gaster and marked with enamel paint^12^. After allowing them to recover for a few minutes, the ants were reinserted into their colonies. After treatment with the stressor, the animals were kept in their housing tubes for 8 days and the survivors were counted every 24 h. Ants were dissected and checked for the developmental status of their ovaries either after death or at the end of the survival assay. We did not observe aversive behaviours towards injected animals until they were dead, thus, premature death caused by aversive behaviours of nestmates can be excluded.

Ants were infected by injecting them with 40 nl of Luria Bertani (LB) medium containing the insect bacterial pathogen *Erwinia carotovora* from an overnight culture that was propagated until reaching an optical density (OD600 nm) of 2. The bacteria were pelleted and taken up in a volume of LB in order to obtain an OD600 nm of 3.6, which was used for injection. Controls were injected with 40 nl of sterile LB medium only. The concentration of bacteria used for injection was chosen from pilot experiments as the concentration that induced mortality after 2–4 days. Colonies and isolation tubes were checked for dead animals every 24 h for 8 days. Because the ant mortality assay contained censored data (i.e., some ants were still alive at the end of the experiment), we applied a Cox regression analysis (SAS version 9.0: Proc PHREG) to test for the effect of ‘life-history stage’ on survival of the ants.

Ants were injected with 40 nl of Schneider’s *Drosophila* medium containing 500 mM paraquat as the experimental treatment or 40 nl of sterile Schneider’s only. The dose was chosen based on pilot experiments in order to induce death in 2–5 days. The paraquat solution was prepared immediately prior to the injection procedure and used within 15 min. Observation procedures, sample sizes and statistical analysis were the same as those described above for the infection experiments.

### Quantification of enzyme activities

To elucidate whether enzymatic antioxidants are responsible for the differing stress resistances and life expectancies observed in the *Harpegnathos saltator* groups, we quantified the activities of systems involved in reactive oxygen species ROS detoxification. Two groups of enzymes are central to ROS detoxification: 1) catalase and superoxide-dismutase (SOD), and 2) enzymes involved in glutathione metabolism, namely glutathione-S-transferases (GST) and glutathione peroxidases (GPx). Levels of reduced (GSH) and oxidized glutathione (GSSG) were measured as well. We used non-manipulated workers and those scored as being aggressive for the determination of enzymatic activities and glutathione contents.

Ants were killed by freezing at −80 °C. Subsequently, each ant was homogenized in liquid nitrogen, resuspended in 110 μl of cold 1x PBS and vortexed. To remove debris, samples were centrifuged at maximum speed in a tabletop centrifuge at 4 °C for 5 min. The samples were then frozen at −80 °C in aliquots of 50 μl until further use. Determination of enzymatic activity was usually performed with 10 samples handled independently.

The enzyme assays were carried out as follows. Superoxide-Dismutase. SOD Activity was determined according to the methods of Marklund and Marklund^28^, which is based on the inhibition of autoxidation of pyrogallol by SOD. Catalase. The method of Johansson and Borg^29^, which measures the production of formaldehyde from a hydrogen donor (methanol) with the chromogen purpald, was used to measure catalase. Glutathione-S-Transferase. The GST activity measurements were conducted as described previously^30^. In this assay, the rate of glutathione conjugation to the substrate CDNB (1-chloro-2,4-dinitrobenzene) is measured. Glutathione-peroxidase activity. GPx activity was measured according to the method of Lawrence and Burk^31^, which utilizes a coupled enzymatic assay, beginning with the reduction of cumene hydroperoxide. Glutathione-content. The total glutathione content, as well as the oxidized and reduced glutathione, were quantified according to well established methods^32^,^33^. If not stated otherwise, chemicals were purchased from Carl Roth (Karlsruhe, Germany) or Sigma-Aldrich (Deisenhofen, Germany).

### Statistical analyses

The statistical evaluation of the survival assay data (infection with *Erwinia carotovora* and injection of paraquat solution) was carried out by using the Cox regression analysis (SAS version 9.0: Proc PHREG). The number of animals per experimental group was n = 20. The data concerning the level of glutathione and the activities of the various enzymes were treated as following: The arithmetical means and the standard errors of each group were calculated. Further statistical evaluation was carried out using SPSS Version 13.0. To test whether a statistical difference to the control group exists, a one-way ANOVA (analysis of variance) and a Levene-test for homoscedasticity was used. If homoscedasticity was found, the Dunnett-test was applied. If homoscedasticity was not on hand, the test after Games-Howell was carried out. Gaussian distribution was verified with Kolmogorow-Smirnow and Shapiro-Wilk. In case of non-Gaussian distribution, the data were ln-transformed and the non-parametric Mann-Whitney-U-Test was applied. Numbers of independent samples are listed in the figure legends.

## Additional Information

**How to cite this article**: Schneider, S. A. *et al.* Social stress increases the susceptibility to infection in the ant *Harpegnathos saltator*. *Sci. Rep.* **6**, 25800; doi: 10.1038/srep25800 (2016).
